# Substandard and Falsified Medicines in Myanmar

**DOI:** 10.3390/pharmacy8010045

**Published:** 2020-03-19

**Authors:** Mirai Sakuda, Naoko Yoshida, Takashi Takaoka, Tomoko Sanada, Mohammad Sofiqur Rahman, Tsuyoshi Tanimoto, Theingi Zin, Kazuko Kimura, Hirohito Tsuboi

**Affiliations:** 1 Institute of Medical, Pharmaceutical and Health Sciences, Kanazawa University, Kanazawa 920-1192, Japan; saku0220@stu.kanazawa-u.ac.jp (M.S.); naoko@p.kanazawa-u.ac.jp (N.Y.); dmpc14@p.kanazawa-u.ac.jp (T.T.); tmk810@stu.kanazawa-u.ac.jp (T.S.); 2 Medi-Quality Security Institute, Graduate School of Medical Sciences, Kanazawa University, Kakuma-machi, Kanazawa 920-1192, Japan; rahmansofique@gmail.com (M.S.R.); kimurak@p.kanazawa-u.ac.jp (K.K.); 3 Pharmaceutical and Medical Device Regulatory Science Society of Japan, Osaka 150-0002, Japan; tsuyoshi-tanimoto@pmrj.jp; 4 Department of Food and Drug Administration, Naypyidaw 15000, Myanmar; zintheingi9@gmail.com

**Keywords:** falsified medicines, substandard medicines, Myanmar, authenticity, distribution

## Abstract

**Background:** substandard and falsified medicines (SFMs) are a threat to public health. The availability of SFMs in Myanmar was reported by the World Health Organization (WHO) in 1999, but there have been few systematic surveys on falsified medicines in Myanmar since then. The aim of this study is to examine the extent of SFMs for sale in Myanmar. **Methods:** target medicines were tablets of candesartan, metformin, and pioglitazone, and infusions of ciprofloxacin and levofloxacin. Samples were collected from hospitals, pharmacies, and wholesalers located in the Mandalay region in 2015. We carried out observation testing, authenticity investigation, and quality testing to search for SFMs, and analyzed the relationship between SFMs and the price and store type. **Results:** There were no falsified medicines found in the authenticity check, though there remained a problem due to low response rates from manufacturers and regulatory authorities. In the quality test, some tablets of metformin and pioglitazone made in India failed the dissolution test. **Conclusions:** although no serious problems were found, some substandard medicines were detected. Regular surveys to monitor SFMs are therefore recommended, together with further regulatory guidance to improve conditions in all medicine manufacturers, distributors, and pharmacies.

## 1. Introduction

Substandard and falsified (SF) medical products are a threat to public health, and may lead to increased mortality and morbidity, as well as emerging drug resistance [1]. We previously investigated SF products, focusing on SF medicines (SFMs), especially in pharmacies and wholesalers in Cambodia. SFMs were detected due to unsanitary storage conditions, poor adherence to Good Manufacturing Practice (GMP) in the pharmaceutical industries, or illegal production of medicines. For example, we found unacceptable amoxicillin and clavulanic acid tablets during quality testing, as well as metformin extended-release tablets, which dissolved much too quickly [2,3,4]. In addition, some cefixime tablets showed spelling errors on the cartons [2,3,4]. Although Myanmar is located near Cambodia, research activities were restricted until political reforms were undertaken in 2011 [5]. Exceptionally, the World Health Organization (WHO) reported in 1999 that 15% of medicines in Myanmar were SFMs [6]. Further, there were sporadic reports concerning Myanmar in 2001 and 2008 [7,8]. Therefore, we started a survey in Myanmar in 2014 [9,10], and followed that with the present in 2015. Because the number of patients with long-term diseases in developing countries is increasing [11], not only on anti-microbials, but also on medicines against chronic or life-style related diseases are important.

In our previous survey [9], pharmaceutical shops in Yangon were equipped with awnings to protect medicines from direct sunlight, and 39.2% were equipped with air conditioning. The average temperature and the humidity in air-conditioning equipped/non-equipped locations were 30.8 ± 2.16 °C/28.6 ± 2.64 °C, and 69.3 ± 8.70%/67.9 ± 12.37%, respectively. In some cases, motorbikes, dogs, or cats were observed in the dispensing area of shops.

Ciprofloxacin infusion (CPFX) and levofloxacin infusion (LVFX) are broad-spectrum new quinolone antibiotics active against both Gram-positive and Gram-negative bacteria. The reason we investigated these injected products is that the Food and Drug Administration, Myanmar (MFDA) informed us that some contaminated antibiotics infusions had been previously found. Candesartan (CA), which is an angiotensin receptor blocker used mainly for the treatment of high blood pressure, was examined. Metformin (MF) and pioglitazone (PG), which are medications used to treat type 2 diabetes mellitus (DM), were assessed as medication against life-style related diseases. We previously found dissolution problems in controlled release tablets without sustained release of MF in Cambodia (data not shown). The above-mentioned medicines appear in the Myanmar Essential Medicines List [12]. 

This study aims to examine the extent of SFMs sold in Myanmar, focusing on the quality of medicines actually sold to patients. It is relevant to elucidate the distribution and storage conditions, as well as issues in the manufacturing process of medicines.

## 2. Materials and Methods 

This field survey was conducted according to a standard field survey guideline [13].

### 2.1. Sampling Areas

Sampling was carried out in Mandalay, the second most populous city of Myanmar after Yangon with a population of nearly 1.2 million; it is the major trading and communications center for northern and central Myanmar. We selected this city after the 2013’ investigation in Yangon [9]. The sampling sites were private hospitals, clinical pharmacies, community pharmacies and wholesalers in Chan-Aye-Thar-San Township, Pyi-Gyi-Tagon Township, Aung-Myay-Thar-San Township, Mahar-Aung-Myay Township of Mandalay District, Pyin-Oo-Lwin Township of Pyin-Oo-Lwin District, and Mite-Htee-Lar Township of Mite-Htee-Lar District in the Mandalay region. The sampling was conducted during 30 September to 6 October 2015. The sampling areas were selected according to the MFDA information as to where regional hospitals often dispense medicine without any prescriptions.

### 2.2. Sample Collection

The principal targets were CA 8 mg tablets, CPFX 200 mg/100 mL infusions in bottles, LVFX 500 mg/100 mL infusions in bottles, MF 500 mg immediate release tablet (MF-IR) and extended- release tablets (MF-ER), and PG 15 mg and 30 mg tablets. In addition to hospitals and wholesalers in the community, community pharmacies, clinical pharmacies in clinics, and wholesalers with community pharmacies were visited along the streets leading to hospitals and wholesalers.

Medicines were purchased according to the following procedures. Products of each target medicine were arranged in the order of price at each shop. According to random number tables, as many products as possible for each medicine were purchased at a shop, but only up to three products for each medicine. The same sample code was assigned to each sample of the same product with the same batch number collected at the same shop at the same time.

Two teams were trained for sampling. Each team consisted of a local supervisor, a local assistant, and one or two researchers from Japan. Information such as the product name, strength, dosage form, manufacturer, wholesaler, manufacturing date, and expiry date was collected on a sampling form, which was completed immediately after the purchase of each sample. Samples were sealed in a plastic bag and a serial number label was attached immediately after the collection. Vehicles and the storage room were air-conditioned and shielded from sunlight. As a principle, 100 dosage units for tablets and 10 dosage units for infusions were collected.

### 2.3. Observation Testing

The environment sanitation and storage conditions, such as temperature and humidity, were examined and noted.

The description and condition of samples were observed carefully with the help of the “Tool for Visual Inspection of Medicines” produced by the International Council of Nurses in partnership with the United States Pharmacopoeia (USP) and modified by the Military and Emergency Pharmacists Section of the International Pharmaceutical Federation [14]. In addition, we observed containers for insects or insect damage, legibility of regulatory codes, etc., misaligned seals, and holes.

### 2.4. Authenticity Investigation

An authenticity investigation was performed according to the modified WHO method [6]. The procedure was to ask the manufacturers (listed on the label of the product) about the product authenticity, and also to ask the Medicines Regulatory Authorities (MRA) of the manufacturing countries about the legitimacy of the products and manufacturers. Questionnaires, including pictures of the sample and, if requested, some tablets, were sent to the all the manufacturers and MRAs for evaluation of authenticity. Questionnaires were sent to the respective manufacturer of each medicine by e-mail in February 2016. Registration numbers in Myanmar were verified by the MFDA.

### 2.5. Quality Analysis

For tablets, we conducted identification (ID), quantity (QT), content uniformity (CU), and dissolution (DS) assessments, and for infusions, we conducted QT, bacterial endotoxin, and sterility assessments, pH determination, and osmolarity determination on the basis of the pharmacopoeia cited on the packaging of the medicines, i.e., the USP, British Pharmacopoeia (BP), or Japanese Pharmacopoeia (JP) [15,16,17], as well as the reported methodology [18]. The results of sterility testing were confirmed by Japan Food Research Laboratories. The quality was evaluated according to pharmacopeial criteria. In some samples, the final judgement was pending because insufficient samples were available. 

### 2.6. Statistical Analysis

Statistical analysis was performed using the *t*-test, the Wilcoxon rank-sum test, and Fisher’s exact test in the Japanese version of International Business Machines (IBM) SPSS Statistics 25 (IBM Japan, Tokyo, Japan). The criterion of statistical significance was set at 5%. Data are presented as mean ± standard deviation (SD).

### 2.7. Ethics

The implementation of this sampling was approved by the Myanmar government. This article is exempt from ethics review in Kanazawa University. This article does not contain any studies with human or animal subjects performed by any of the authors.

## 3. Results

### 3.1. Sampling Collection

#### 3.1.1. Sampling Sites

A total of 219 samples were purchased from 91 pharmacies, consisting of 22 private hospitals, 7 clinical pharmacies, 46 community pharmacies, 12 wholesalers with community pharmacies, and 5 wholesalers in six townships in the Mandalay region (Table 1). All hospitals and pharmacies that were visited participated in sampling.

#### 3.1.2. Samples

**Figure 1:**
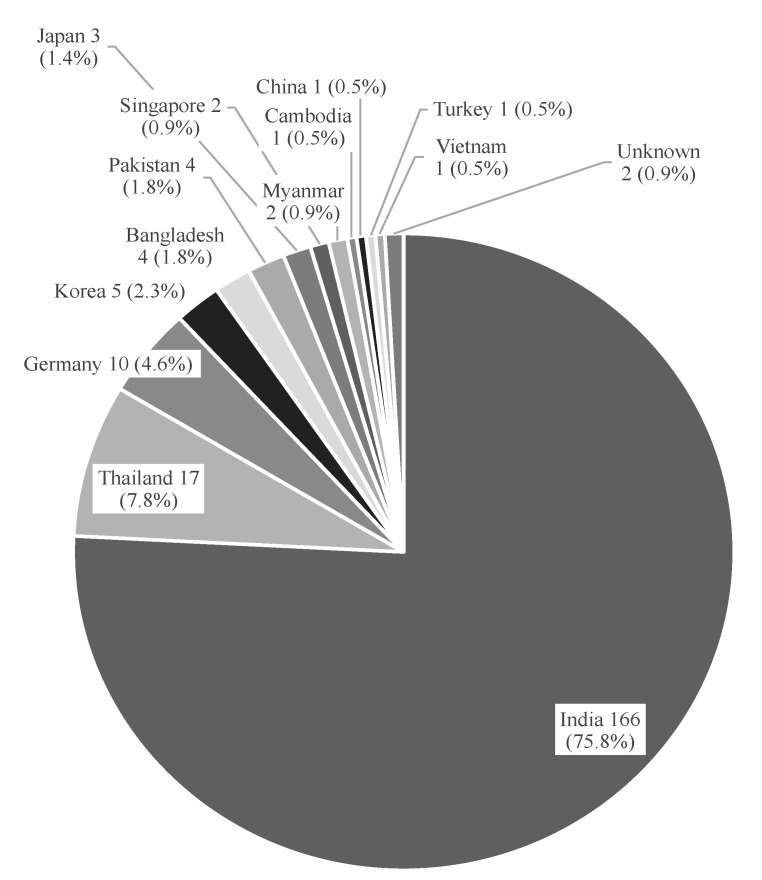
The result of sample collection. The graph shows the number of samples, the country of manufacture, and the proportion of the total.

The results of sample collection are summarized in Table 1. We collected 219 samples manufactured by 49 manufacturers in 13 countries. Among them, three CA products, two LVFX products, one PG, and one MF-IR product were branded medicines, and the others were generics. The numbers of samples were 10 for three CA products, 45 for seven CPFX products, 42 for eight LVFX products, 62 for 28 MF products, and 60 for six PG products. Among the MF products, 49 samples were MF-IR and 13 were MF-ER. CA samples were collected mainly for the purpose of authenticity investigation. Two samples out of 219 were produced by one manufacturer in Myanmar, and the remaining 217 samples were imported from 48 manufacturers in 12 countries. Out of 219 samples, 166 samples (75.8%) were imported from India and 17 (7.8%) from Thailand (Figure 1).

### 3.2. Observation Testing

#### 3.2.1. Observation of Shops

Some pharmacies had a motorbike, a dog, or birds inside the store, and placed medicines directly on the earth floor. Some outlets put drinks in the refrigerator with medicines.

All shops were equipped with awnings to protect medicines from direct sunlight. On the other hand, 32 out of 91 shops (35.2%) were equipped with air-conditioning. Twenty-nine shops actually operated air-conditioning (average temperature 29.7 ± 1.8 °C, average humidity 65.4% ± 11.1%). 59 shops did not have air-conditioning, and three shops had air conditioning that was not working (average temperature 30.1 ± 2.3 °C, average humidity 70.9% ± 9.9%). There was no significant difference in average temperatures between shops with and without air conditioning, though there was a significant difference in humidity (*t*-test, *p* < 0.05). 

Although labeled storage information instructs that CA, CPFX, LVFX, MF, and PG should be stored below 25 °C or below 30 °C in a dry place, protecting from light and moisture, 98 out of 219 samples (44.7%), were stored at or below the recommended maximum temperature.

#### 3.2.2. Observation of Samples

**Figure 2:**
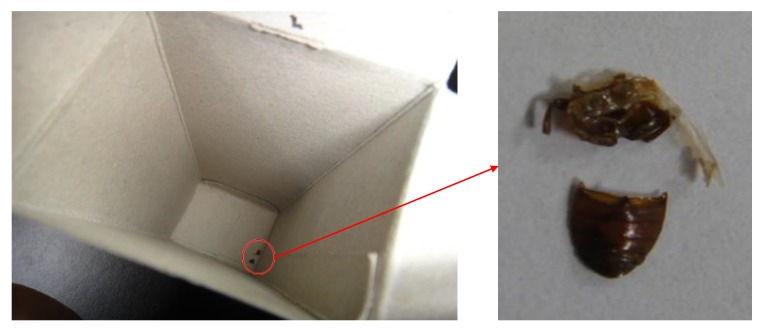
Insect carcasses were detected in an individual packaging.

**Figure 3:**
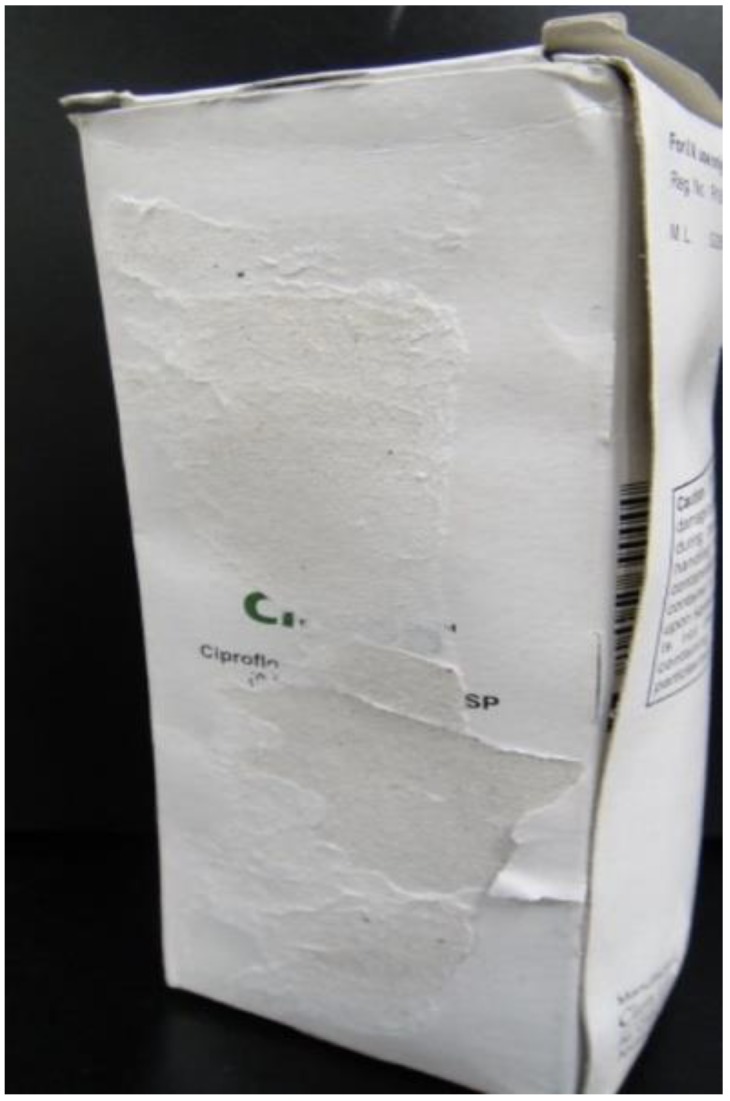
It already had an illegible label when we sampled in Myanmar. Furthermore, the adhesive surface of the container peeled off and it was cracked.

The following issues were identified by observation: The container of one CPFX sample contained insect carcasses (Figure 2). As for PG, a strip from another PG sample was mixed with the regular strips inside the box in one sample. Six samples had no container and three samples had battered containers upon which the labelled information could not be read because the box surface was sticky (Figure 3). Two CPFX samples and one PG sample lacked a Myanmar registration number. One CPFX sample had labelling written in Russian; however, no abnormal tablets or infusions were observed.

### 3.3. Authenticity Investigation

**Figure 4:**
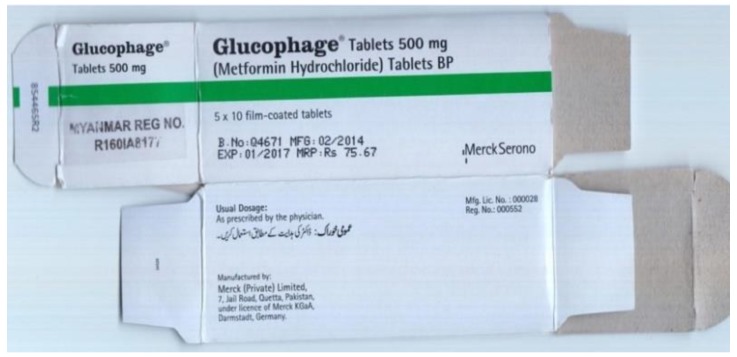
It already had an illegible label when we sampled in Myanmar. Furthermore, the adhesive surface of the container peeled off and it was cracked.

Six manufacturers, including two branded medicine manufacturers (Takeda Pharmaceutical Company Limited and Merck (Private) Limited), replied, covering 19 samples of seven products: 18 samples were confirmed as genuine products and one MF sample was allegedly misbranded. This MF manufacturer stated that the last consignment of its product to Myanmar was sent in 2012, while the manufactured date printed on B-068 was “02/2014” (Figure 4). No reply was received concerning 200 samples. Most manufacturers gave a contact e-mail address, but in most cases, we did not obtain a reply, even after a reminder was sent. 

Thirteen MRAs in manufacturing countries were contacted by e-mail in February 2016, and those in China, Germany, Ireland, Myanmar, and Singapore replied, stating that their six manufacturers and all 16 products were legitimate.

As for registration by MFDA, all samples of CA, LVFX, and MF were registered (100%), while no registration number was found on two CPFX samples, and one PG sample was not registered (Table 2). Unregistered samples were purchased at a private hospital or a wholesaler with a community pharmacy.

### 3.4. Quality Analysis

Out of 219 samples, 218 samples were tested for quality (Table 3). Among the 218 samples analyzed for QT, 27 failed. Of 132 samples analyzed for CU, three were pending. Of 132 samples analyzed for DS, 10 samples were pending and nine samples failed. For PG, eight failed samples were from the same manufacturer. For MF, 1 MF-IR sample out of 49 (2.0%) failed. All samples made in Myanmar passed the quality test. The failed samples across all analysis tests were manufactured in two countries: 1 in Pakistan (out of 4, 25%), and 34 in India (out of 166, 20.5%). 

All samples passed the sterility and endotoxin tests. A total of 42 samples of LVFX passed the foreign insoluble matter, pH, and osmolarity tests.

### 3.5. Factors Influencing the Outcome of the Quality Testing

Quality test results were not related to shop type for sampling, though some samples bought from wholesalers failed some tests (Table 4: Fisher’s exact test, *p* = 0.66). The relation between price and quality is shown in Table 5. There were significant differences in the average prices of passing and failing MF-ER and PG (15 mg). Quality-test-passing PG (15 mg) tended to cost more than the failing products (n = 59, Wilcoxon rank sum test, *p* < 0.05). However, quality-test-failing MF-ER tended to cost almost twice the price of the passing products (n = 8, Wilcoxon rank sum test, *p* < 0.05). There was no significant difference in the average prices between passing and failing CA, CPFX, MF-IR, or LVFX (Table 5).

There was no significant difference between passing and failing medicines in terms of the use of air conditioning at the place of purchase. In addition, there was no relation between temperature at the place of purchase and quality of medicines.

## 4. Discussion

We examined the extent of falsified and substandard medicines in Mandalay, Myanmar. We found that more than half of all samples were not stored at or below the required temperature, and we found some substandard medicines and unregistered medicines. There still appears to be some smuggling of medicines, as well as erroneous formulations leading to dissolution failure. A manufacturer of PG whose samples always failed the quality test did not meet the licensing criteria as a pharmaceutical manufacturer. 

### 4.1. Observations

Natural conditions in Mandalay (average 27.0 °C) [19] are not favorable for storing pharmaceuticals that must be kept below 25 °C, although air conditioning did keep the temperature below 30 °C, which is suitable for the storage of many pharmaceutical products. Only 98 of 219 samples (44.7%) were stored at or below the required temperature. There was almost no temperature difference or quality difference between shops with air conditioning and those without it. Since environmental temperatures were relatively low at the time of this survey, the situation might have been different if the survey had been carried out in a hotter season. In principle, all the shops should have an air conditioner.

We did observe unsanitary environments: a motorcycle was kept inside the store, insect carcasses were found in a box, and some labels could not be read. This could have been due to water damage or exposure to rain, suggesting that some storage facilities need to be improved.

The pharmacy management system has been greatly improved compared to that in 1999, and no unlabeled, repackaged polyethylene bags were on sale. 

### 4.2. Authenticity

Three unregistered samples were detected in Mandalay. These would not have been evaluated for local use, and so consumers should be warned to avoid unregistered medicines. Further education of suppliers is recommended in this regard.

During the authenticity investigation, one MF-IR sample from a manufacturer in Pakistan was claimed to be misbranded, as the last consignment to Myanmar was sent in 2012 and the product was manufactured in 2014. This incident may suggest that there is an unofficial route of export/import from Pakistan to Myanmar. It is established that illegal distribution occurs in developing countries [20]. 

Better cooperation from manufacturers in responding to questionnaires for authenticity investigation remains an important goal.

### 4.3. Quality Analysis

The quality of most medicinal samples collected in Mandalay in 2015 was acceptable. Although falsified medicines were detected in a 2014 study, no falsified medicines have yet been found in the present study. 

In the DS assessment of MF, 1 MF-IR sample out of 49 samples (2.0%) and 9 MF-ER samples out of 13 samples (69.2%) were pending. All the medicines contained an acceptable amount of active ingredient, so the tablets could harden and change in terms of other properties, even though the content may not be lost due, for example, to heat [21,22], since extended release tablets and sustained-release tablets require special technology that may not be readily available to manufacturers in developing countries [23]. Quality-test-failing MF-ER samples were manufactured by one manufacturer out of four. Improvement of the storage conditions (temperature and humidity) within the pharmacies can solve the problem.

For PG tablets, there was a significant difference in the results of dissolution tests between manufacturers. All the PG samples of one manufacturer failed the quality test. This might be due to manufacturing inadequacies [24]; at present, we are still investigating the cause.

Interestingly, some samples bought from wholesalers failed in the quality testing (Table 4), suggesting that the unacceptable tests results were not necessarily due to unsanitary storage conditions in pharmacies, but had already emerged during manufacture or wholesale distribution. 

### 4.4. Price and Falsification

We found that the same products were sold at different prices. Indeed, for standard generic MF-IR, the price difference reached a factor of 13.6. However, the branded MF-IR was cheap, because it was misbranded and mispriced.

Quality-test-failing MF-ER tended to cost almost twice as much as the passing products, whereas quality-test-passing PG (15 mg) tended to cost more than failing products. Thus, high price does not necessarily imply good quality. 

Some falsified medicines were detected at community pharmacies, but the falsification rate was lower and the quality of medicines was higher than those in the 1999 survey in Myanmar. Sampling in Myanmar is valuable and rare, because there was very little up to now. It is worthwhile to carry out sampling as a first step in the quality assurance of pharmaceutical products in Myanmar.

### 4.5. Limitations

There are limitations in the present study. The number of samples, the sampling area, the number of sampled outlets, and the kinds of medicines sampled were all limited in this work. We did not use ideal approaches, such as systematic sampling or random sampling for avoiding bias. Thus, it is not possible to estimate the overall quality of medicines in Myanmar.

## 5. Conclusions

In this study, we did not find any serious problems in collected CA, CPFX, LVFX, MF-IR, MF-ER, and PG in Mandalay, Myanmar. However, some samples did not pass ID, quality testing (QT, CU, DS), some unregistered samples were distributed. In addition, more than half of shops exposed medical products to temperatures higher than recommended temperature. There was an insect in the sample container, and the adhesive surface of a container peeled off and cracked. Our results indicated that there is still some room for improvement in terms of environment sanitation, storage conditions, and distribution chains. Further guidance for medicine manufacturers, distributors, and pharmacies may be needed to ensure rigorous observance of GMP, Good Distribution Practice, and Good Pharmacy Practice.

## Figures and Tables

**Table 1:** Sampling sites of medicines.

Active Ingredient		Candesartan (8 mg)	Ciprofloxacin Infusion (200 mg/100 mL)	Levofloxacin Infusion (500 mg/100 mL)	Metformin IR (500 mg)	Metformin ER (500 mg)	Pioglitazone (15 mg)	Pioglitazone (30 mg)	Total
Number of samples		10	45	42	49	13	59	1	219
Number of shops		8	44	37	51		49		91
Number of samples collected at sampling site	Private hospital (n = 22)	2	17	20	8	6	9	0	62
	Clinical pharmacy (n = 7)	0	2	3	9	0	1	0	15
	Community pharmacy (n = 46)	3	16	12	19	2	34	0	86
	Wholesaler with community pharmacy (n = 12)	4	6	5	9	1	11	1	37
	Wholesaler (n = 5)	1	4	2	4	4	4	0	19

IR: immediate-release tablet, ER: extended-release tablet.

**Table 2:** Registration ratio of collected medicines.

Medicine	Registered	Non-Registered
Candesartan (n = 10)	10 (100%)	0 (0%)
Ciprofloxacin infusion (n = 45)	43 (96%)	2 (4%) *
Levofloxacin infusion (n = 42)	42 (100%)	0 (0%)
Metformin (n = 62)	62 (100%)	0 (0%)
Pioglitazone (n = 60)	59 (98%)	1 (2%)
Total (n = 219)	219 (99%)	3 (1%)

* Two different products.

**Table 3:** Results of quality testing (n = 218).

	Quantity Test		Content Uniformity Test			Dissolution Test			Sterility		Endotoxin	
Medicine	Pass	Fail	Pass	Fail	Pending	Pass	Fail	Pending	Pass	Fail	Pass	Fail
Candesartan (n = 10)	9	1	10	0	0	10	0	0	n.a.	n.a.	n.a.	n.a.
Ciprofloxacin (n = 44) *	38	6	n.a.	n.a.	n.a.	n.a.	n.a.	n.a.	44	0	44	0
Levofloxacin (n = 42) **	28	14	n.a.	n.a.	n.a.	n.a.	n.a.	n.a.	40	0	42	0
Metformin IR (n = 49)	49	0	49	0	0	47	1	1	n.a.	n.a.	n.a.	n.a.
Metformin ER (n = 13)	8	5	10	0	3	4	0	9	n.a.	n.a.	n.a.	n.a.
Pioglitazone (n = 60)	59	1	59	0	1	52	8	0	n.a.	n.a.	n.a.	n.a.
Total	191	27	128	0	4	113	9	10	84	0	86	0

n.a.: not applicable; * One sample was not tested; ** Sterility test was not done on two samples.

**Table 4:** Relation between sampling site and medical quality.

	All Pass	Any Fail	Pending
Private hospital	48	9	4
Clinical pharmacy	12	3	0
Community pharmacy	73	12	1
Wholesaler with community pharmacy	31	6	0
Wholesaler	13	5	1

All pass: the sample passed all quality tests; Any fail: the sample failed at least one quality test; Pending: not included in the other columns.

**Table 5:** Relation between price and medical quality.

		N	Mean ± SD	*p* Value
Candesartan (generic)	All pass	6	0.125 ± 0.0186	n.t.
	Fail	1	0.141	
Ciprofloxacin	All pass	38	0.387 ± 0.177	n.s.
	Fail	6	0.355 ± 0.134	
LVFX (generic)	All pass	26	2.42 ± 0.435	n.s.
	Fail	14	2.20 ± 0.368	
Metformin IR (generic)	All pass	46	0.0383 ± 0.0254	n.t.
	Fail	2	0.0430 ± 0.0387	
Metformin ER	All pass	3	0.0324 ± 0.0116	<0.05
	Any fail	5	0.0559 ± 0.0128	
Pioglitazone (15 mg)	All pass	51	0.0709 ± 0.0152	<0.05
	Any fail	8	0.0550 ± 0.0079	

Prices are expressed in U.S. dollars (1 Kyat = 0.00078 US dollars (as at 29 September 2015)). Pending samples are not included; n.s.: not significant, n.t.: not tested. LVFX = levofloxacin infusion.
